# A dual-stream model based on PRNU and quaternion RGB for detecting fake faces

**DOI:** 10.1371/journal.pone.0314041

**Published:** 2025-01-28

**Authors:** Mohan Hua, Shuangliang Li, Jinwei Wang

**Affiliations:** 1 Nanjing Foreign Language School, Nanjing, China; 2 Nanjing University of Information Science and Technology, Nanjing, China; Khalifa University of Science and Technology, UNITED ARAB EMIRATES

## Abstract

The forensic examination of AIGC(Artificial Intelligence Generated Content) faces poses a contemporary challenge within the realm of color image forensics. A myriad of artificially generated faces by AIGC encompasses both global and local manipulations. While there has been noteworthy progress in the forensic scrutiny of fake faces, current research primarily focuses on the isolated detection of globally and locally manipulated fake faces, thus lacking a universally effective detection methodology. To address this limitation, we propose a sophisticated forensic model that incorporates a dual-stream framework comprising quaternion RGB and PRNU(Photo Response Non-Uniformity). The PRNU stream extracts the “camera fingerprint” feature by discerning the non-uniform response of the image sensor under varying lighting conditions, thereby encapsulating the overall distribution characteristics of globally manipulated faces. The quaternion RGB stream leverages the inherent nonlinear properties of quaternions and their informative representation capabilities to accurately describe changes in image color, background, and spatial structure, facilitating the meticulous capture of nuanced local distinctions between locally manipulated faces and real faces. Ultimately, we integrate the two streams to establish the exchange of feature information between PRNU and quaternion RGB streams. This strategic integration fully exploits the complementarity between two streams to amalgamate local and global features effectively. Experimental results obtained from diverse datasets underscore the advantages of our method in terms of accuracy, achieving a detection accuracy of 96.81%.

## Introduction

**Fig 1:**
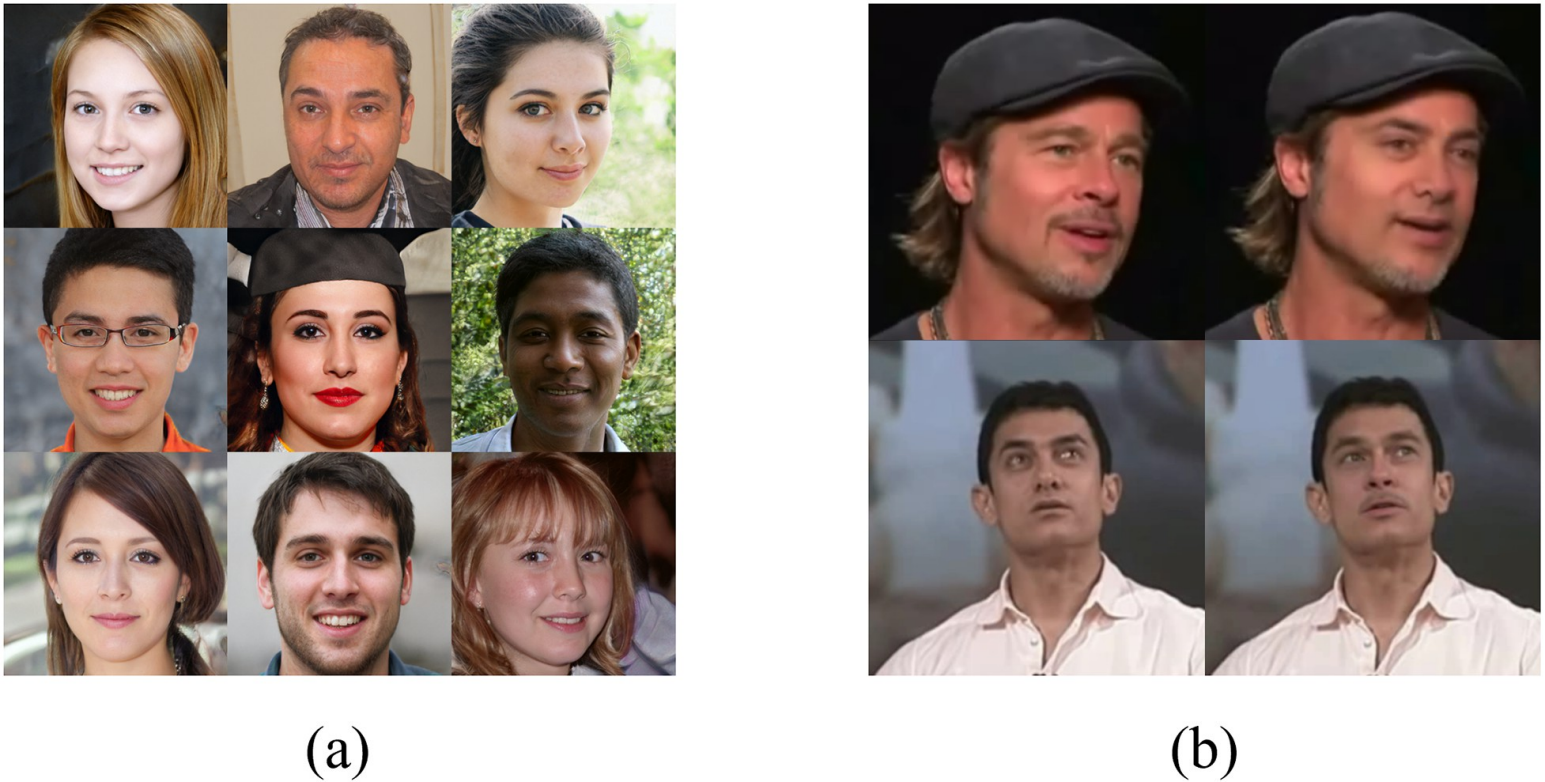
Fake faces generated by StyleGAN and Deepake, where (a) represents the fake faces in the StyleGAN dataset and (b) portrays face-swapping images created by Deepfake. The first column represents real faces, while the second column depicts the manipulated faces.

With the advancement of AIGC(Artificial Intelligence Generated Content) [1], fake faces are becoming increasingly sophisticated and realistic, leading to a frequent occurrence of deceptive incidents that profoundly impact societal security. Common global facial manipulation techniques, such as StyleGAN [2] is capable of generating high-quality fake faces. StyleGAN is a type of Generative Adversarial Network(GAN) [3] that generates realistic images at very high resolutions, capable of producing high-quality, lifelike faces, including those with intricate textures and rich expressions. This technology blurs the boundary between fake and real faces, making it difficult to discern their authenticity with the naked eye. Deepfake [4, 5] is a local facial manipulation techniques that applies one person’s facial features onto the image or video of another person, creating highly deceptive and misleading fake faces. Fig 1 illustrates the fake faces generated by StyleGAN and Deepake, highlighting the highly deceptive nature of the fake faces produced by AIGCs, making them difficult to discern with the naked eye. With the advancement of fake face technology, the abuse and manipulation of such technology in social media, identity authentication, and various security applications have become increasingly challenging to identify and counteract. This poses a significant challenge to the credibility and authenticity of societal information.

In the face of this issue, researchers strive to seek reliable technical means to differentiate between authentic and fake faces, ensuring the protection of information security and personal privacy. In this context, many effective facial forensic algorithms have been proposed. Based on the distinction in feature processing, we categorize forensic methods into traditional image forensics-based approaches [6–8] and deep learning-based approaches [9–14]. Traditional image forensics methods focus on analyzing the physical properties, structures, and textures of faces. They involve meticulously designed feature extraction and image processing steps to detect fake faces. Nataraj et al. [9] introduced a pixel-level fake image detection method into fake face detection, using color co-occurrence matrices as inputs. McCloskey et al. [8] identified differences in image color processing between real and AIGC images, attempting to use Support Vector Machine(SVM) [15] for the final classification. However, the experimental results showed significantly lower accuracy than expected. Deep learning-based methods utilize deep neural network models to learn the underlying feature differences between fake and real faces. Wang et al. [10, 11] integrated traditional features with convolutional neural networks to construct a dual-stream network architecture for detecting human faces generated by StyleGAN, achieving promising results. This mechanism assists the network in better focusing on the subtle feature differences between real and fake faces, leading to more accurate classification. Zhang et al. [13] combined traditional forensic techniques with deep learning, utilizing accelerated robust features and bag-of-words techniques to extract image features. They then employed SVM, Random Forest(RF) [16], and Multilayer Perceptron(MLP) [17] for the classification discrimination between real and fake faces.

While the existing methods achieve relatively high accuracy in detecting fake faces, they are tailored to fake faces generated by a single technology. When faced with various types of fake faces generated by diverse technologies, the adaptability and generalization capabilities of these models are greatly reduced. In current research, there is no comprehensive solution to the detection of globally and locally manipulated faces. This implies that, despite the substantial achievements in recognizing fake faces generated by a single technology, there is a need to further enhance the robustness and generalization of the models in practical applications, to address the increasingly complex and diverse challenges posed by fake face forgery technologies.

To overcome this challenge, we propose a fake face detection model based on global and local feature fusion, which can detect fake faces through global and local operations. For fake faces with global operations, we integrate PRNU features with the network to design a PRNU feature stream. By utilizing the features of automatic feature learning in deep learning methods, we aim to learn more complex and representative feature mappings, to overcome the lack of adaptability of traditional methods to fake faces generated by advanced AIGC. Different from the conventional PRNU extraction method, we choose to use Haar wavelet to extract PRNU [18]. The PRNU features extracted by conventional methods are too simple, which is sufficient for the feature characterization of fake faces, and the performance is poor in practical application. Haar wavelet transform has the adaptability of scale and direction, and it decomposes the image into low-frequency parts and high-frequency parts in different scales and directions, which can effectively capture the high-frequency noise of PRNU. It can also capture fake signs of fake faces in non-smooth areas such as features and facial contours. For fake faces with local tampering, we propose a quaternion RGB stream. Quaternion CNN can process RGB color channels closely, effectively capture the interdependence between color channels, build a comprehensive face local tampering region information, and enhance the attention of network to local manipulation features. Thus, the subtle color inconsistencies in local tampered faces can be effectively detected. Finally, by making full use of the complementarity of the two streams through feature fusion, the shortcomings of each stream are compensated, thus improving the robustness and accuracy of the network, making the network more comprehensive and able to cope with a variety of possible global and local fake face operations.

The main contributions of this study are summarized below:

- We propose a dual-stream network that integrates PRNU features and quaternion RGB features for the detection of fake faces, enhancing both performance and generalization capabilities in detection.
- By using neural networks to learn the traditional PRNU features end-to-end, the global differences between fake and real faces are captured effectively, overcoming the limitations of artificial design feature extraction methods.
- By constructing quaternion RGB streams, the rich features of faces are extracted from high-dimensional space, which enhances the capture and analysis of subtle local differences between real faces and fake faces.

The rest of this paper is organized as follows. Section 2 discusses the related work. Section 3 provides a detailed description of our detection method. Section 4 presents the experimental results, demonstrating the effectiveness of the proposed method in facial forgery detection. Finally, Section 5 concludes the paper and discusses future research directions.

## Related work

### Fake face generation technique

Based on the different generation technologies, fake faces can be categorized into globally and locally manipulated faces, such as globally manipulated fake faces generated by GANs and locally manipulated faces created by Deepfake. GAN [3] is a deep learning model that uses random noise to generate diverse images, significantly improving the sampling of high-dimensional, complex, and real-world distributions. It has achieved significant success in image synthesis [19] and has become one of the most popular and extensively researched areas in applied studies. Recent advancements such as PGGAN [20] and StyleGAN [2] have made significant progress, generating more realistic images. Kim et al. [21] proposed a dual conditional face generator based on a diffusion model, which is used to consistently generate face images with the same theme in different styles, making the generation more efficient and convenient. Deepfake has a wide range of applications in the field of face images [4, 5], typically employed for localized manipulation such as facial editing, replication, and replacement in several key areas. On the basis of StarGAN [22], Zhang et al. [23] used the method of local perturbation generation to make a fake face, and the generated image was more deceptive. Facial editing such as StarGAN [22], attGAN [24] and STGAN [25] allow the addition, deletion, modification, or alteration of certain attributes of human faces, such as hair, age, etc. Facial replication allows for altering facial expressions while maintaining a sense of facial authenticity. Facial replacement [26, 27] is the process of converting a face from a source image to a target image by taking into account factors such as face size, pose, and skin color of the source and target images.

### Fake face detection technology

With the increasing realism of fake faces, research on detecting fake faces has become a hot research field. Traditional methods rely on manually setting rules and algorithms to analyze facial feature differences and distinguish between real and fake faces. McCloskey et al. [9] utilized color information for detecting fake faces. Wang et al. [7] found significant differences in color processing between generated images and natural images, and proposed a detection scheme based on image color features. By analyzing the differences in appearance between fake and real faces, accurately distinguish between real and fake faces.

Compared with traditional methods, neural networks can capture richer and more complex feature information in images, thus showing stronger robustness and adaptability when confronted with fake faces. Li et al. [12] proposed a fake face detection method based on CNN. They modified the CNN architecture and trained it using supervised learning on a dataset of facial samples. However, this custom CNN architecture exhibits relatively weak robustness and is vulnerable to attacks from adversarial examples. Nataraj et al. [8] solved this problem by extracting the co-occurrence matrix from each color channel of the image. They combined the resulting features and used CNN to classify and detect fake faces. However, this method dealt with the co-occurrence matrix alone and ignored the color correlation between multiple channels, which had a certain impact on the detection performance. Liu et al. [28] introduced Gram-Net, a new architecture that utilized global image texture representation for fake face detection. Gram-Net is robust to image transformations such as sampling, compression, blurring, and noise. Yang et al. [29] trained a classifier by extracting features that differ between the facial landmark positions of real and fake faces. However, as fake face technology continues to evolve and improve, especially with the introduction of larger data sets, more complex model architectures, and more effective training methods, fake faces are becoming more realistic. Detection faces significant challenges and requires the development of more powerful, intelligent and robust detection technologies to ensure the authenticity and trustworthiness of faces.

### Distribution characteristics of faces

This section primarily analyzes the differences between real and fake faces from two aspects: global PRNU features and local tampering features in deepfake images. These analyses lay the theoretical foundation for the proposed methodology.

#### PRNU characteristics of faces

**Fig 2:**
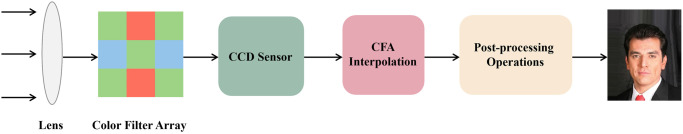
The formation process of a natural image: Natural light passes through a lens, and a color filter array selectively filters light waves of specific frequencies to collect color information of the image. The image sensor converts the transmitted light waves into electrical signals, which are then subjected to post-processing, resulting in the final formation of a natural image.

There are significant differences in the imaging process between fake face and real face. As shown in Fig 2, the imaging process of natural images [30–32] is affected by the accuracy of optical elements and natural lighting conditions, which often leads to various complex manufacturing defects. These defects and the inevitable errors in the manufacturing process lead to the creation of sensor pattern noise, also known as “camera fingerprint”. Because of the uniqueness of PRNU, it is widely used in the recognition and authentication of various real images [33].

The fake face bears no resemblance to the face taken by the camera device. Compared with real faces, fake faces are inferred and generated based on data samples and patterns [32], not directly captured from real scenes, and lack the consistency of real world context that real faces have. As a result, they lack the subtle changes and noise that are present in real faces. In addition, the influence of random factors and learning rules in the generated model further highlights the personalized features of the generated faces, which are manifested by specific texture styles, color deviations and shape changes. Therefore, these traces can be regarded as fingerprints [33, 34] of fake faces, providing valuable clues for the analysis of its generation process and characteristics.

#### RGB characteristics of faces

In the RGB domain, there are subtle yet crucial visual differences between fake faces and real faces [33, 34]. These differences include abnormal color distributions, inconsistencies in texture details, and blurriness or distortion in edge regions. Although fake faces exhibit overall realism, they often lack the intricate textures and natural colors found in real images. In comparison, the real faces typically display more natural, uniform, and subtle color gradients. Especially in terms of high resolution or fine details, fake faces lack visual consistency with real faces.

#### Quaternion and hypercomplex theory

Quaternion, as a kind of hypercomplex number, has received extensive attention in signal processing, computer vision, robot control, and other fields. Especially in the field of neural networks, quaternion neural networks [35, 36] can more accurately capture and analyze the internal structure of data by their unique advantages in processing complex information such as three-dimensional rotation and direction, thus achieving better performance. The latest research [37] comprehensively discusses the cutting-edge applications of hypercomplex neural networks in spatiotemporal data processing and introduces the related concepts, network components, and learning methods of hypercomplex in detail.

## Proposed method

Current research primarily focuses on the isolated detection of globally and locally manipulated fake faces, lacking generalizability in practical applications. To address this issue, we propose a dual-stream CNN detection model to detect fake faces generated by both StyleGAN and Deepfake. By combining PRNU and quaternion RGB features through dual-stream fusion, our method realizes the detection of fake faces with global and local manipulations.

**Fig 3:**
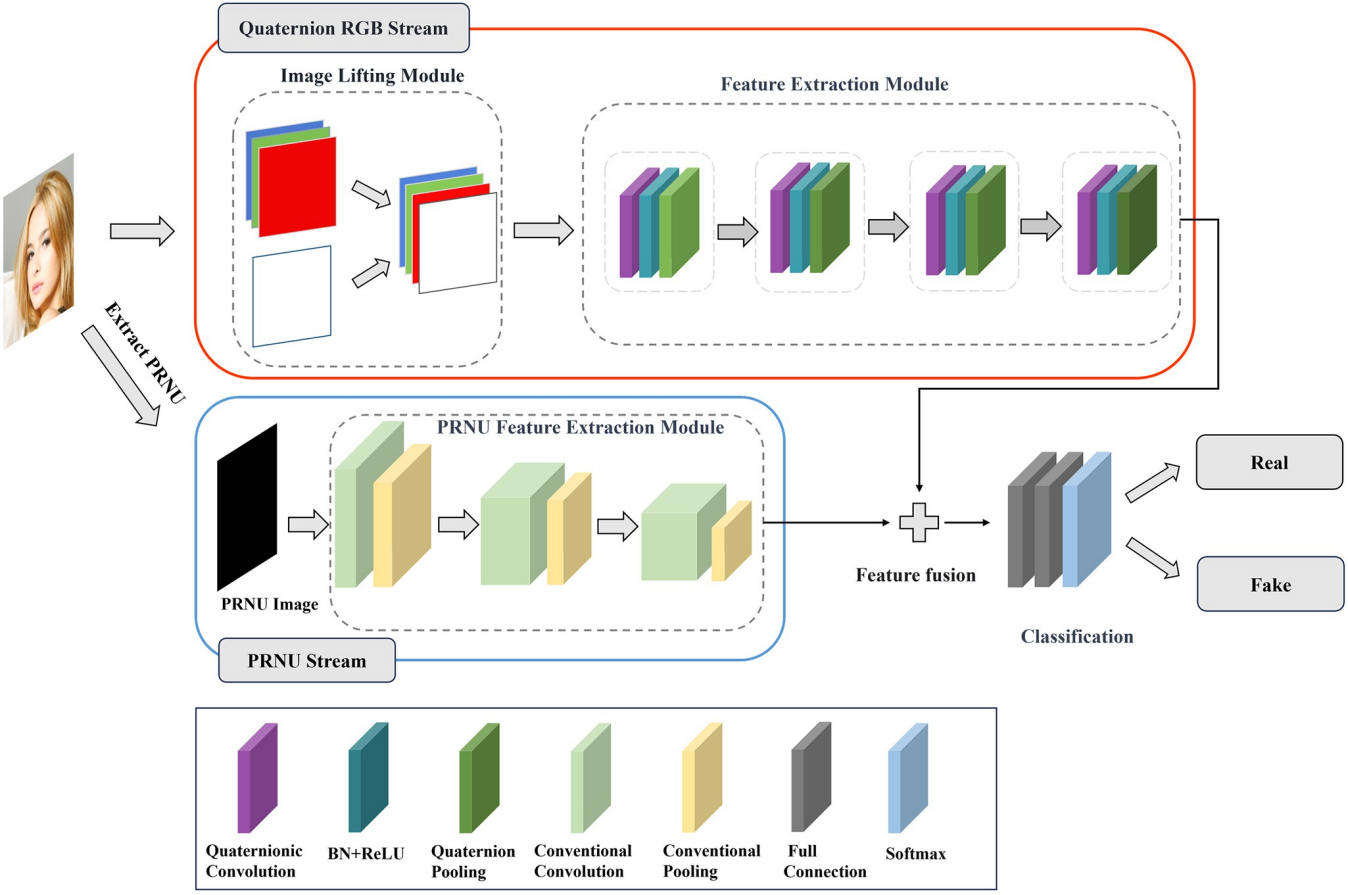
AIGC face detection model based on quaternion RGB and PRNU dual stream drivers (red box represents the quaternion RGB stream network, blue box represents the PRNU stream network).

The schematic diagram of the dual-stream detection model is illustrated in Fig 3. The blue box represents the PRNU feature stream, which includes the global extraction of PRNU noise and network stream construction. This stream fully extracts the global information of faces and learns the global feature difference between real and fake faces, which is used to detect globally manipulated faces. The red box represents the quaternion RGB stream, which utilizes the algebraic properties of quaternions. This stream exhibits low sensitivity to unmodified regions of the image, while effectively amplifying the differences between locally manipulated facial areas, employed for the detection of locally manipulated faces.

### The PRNU stream network

To detect globally manipulated faces, we combine the PRNU features inherent in the camera sensor with a CNN stream, using the network to process the PRNU features end-to-end, aiming to learn more complex and representative feature mappings to detect fake faces. In this approach, CNN utilizes the multiple convolutional layers and pooling layers to extract rich feature representations from the original image. By inputting PRNU features into the CNN network, the network can automatically learn the complex relationships between PRNU features and transform them into higher-level and more discriminative feature representations.

#### Extraction of PRNU

PRNU is composed of high frequency pixel non-uniform noise(PNU) and low-frequency defects. Specifically, PNU refers to the minute and uniformly distributed noise generated by inherent defects in the camera sensor during the imaging process. Low-frequency defects concentrate on the low-frequency components of the image, and do not contain unique characteristics of the camera sensor. Therefore, when extracting PRNU features, low-frequency defects are considered as irrelevant additional noise. They are typically removed through preprocessing steps to more effectively extract and analyze the PNU component. The extraction of PRNU in this paper refers to the extraction of PNU.

The existing research describes image noise in a fundamentally similar manner. Building upon the work of Chen et al. [38], we further refined the image noise model. We formalized the generation process of image pixels in Eq 1,

$$\begin{array}{c} I=(1+K)\cdot O+\sum_{i=1}^{n}A_{i}+n \end{array}$$ (1)

where *I* is the observed pixel value of the image, *O* represents the ideal pixel value, *K*⋅*O* is the multiplicative noise, *K* is the multiplicative noise coefficient indicating the impact of multiplicative noise on the ideal pixel value, *A*_*i*_ represents different types of additive noise to simulate additional noise from different sources in the image generation process, and n represents other unlisted additional noise components.

As a type of high-frequency multiplicative noise, the acquisition process of PRNU is affected by the complex nonlinearities within the camera, making it challenging to obtain directly. When dealing with multiplicative noise, filtering is a common technique. Filtering not only filters PRNU multiplicative noise but can also be combined with other image processing methods such as feature extraction, data analysis, thereby enhancing the overall capability to acquire and analyze PRNU noise. The filtration process is shown in Eq 2,

$$\begin{array}{c} X=I-F(I) \end{array}$$ (2)

where *X* represents the pattern noise, *I* is the pixel value of the image, and *F*(⋅) denotes the filtering operation.

From Eq 2, it can be observed that the extraction of PRNU depends on the choice of F(·). The nonlinear nature of multiplicative noise makes traditional linear filtering less effective in handling multiplicative noise. To address multiplicative noise, we use nonlinear wavelet filtering to extract PRNU noise. The wavelet transformation facilitates multiscale analysis, effectively decomposing and processing various frequency components of the image. This allows for a more effective capture of the multiscale features of PRNU, enabling a comprehensive representation and extraction of global information from faces. This includes overall illumination distribution, texture information, and the overall appearance features of the face. Analyzing and comparing this global information allows for the detection of overall feature anomalies in fake faces, providing crucial clues and evidence for the detection and identification of fake faces.

**Fig 4:**
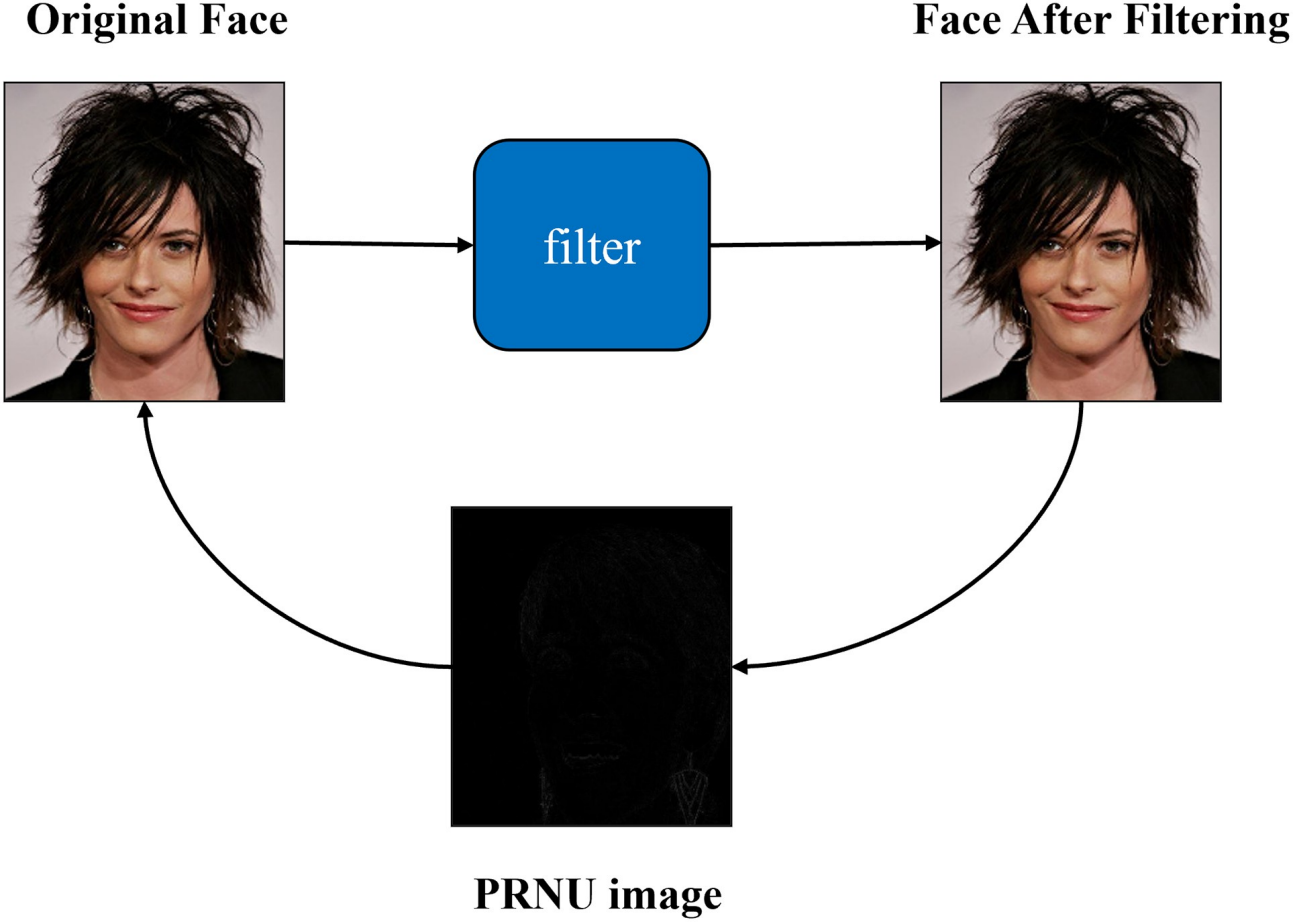
PRNU extraction through image denoising using Haar wavelet basis.

We employ the Haar wavelet basis for image filtering. The conventional camera mode noise extraction method [30] extracts the mode noise through the method of image noise reduction, which focuses on the difference between digital images under different camera sensors and physical conditions such as temperature and illumination and is used for camera traceability of images. However, in the false human detection task, our research focuses on extracting valid feature differences between real and fake faces. Although the traditional method can extract PRNU features effectively, the features extracted by this operation are too simple, which is sufficient for the feature characterization of fake faces, and the performance is poor in practical application. Haar wavelet transform has the adaptability of scale and direction. It can decompose the image into the low-frequency part and high-frequency part in different scales and directions, which can effectively capture the high-frequency noise of PRNU, and also capture the forged signs of fake faces in non-smooth areas such as facial features and facial contours. We perform two haar wavelet transforms on the image to obtain a low-frequency component (LL) and three high-frequency components (HL, HH, and LH), then zero the high-frequency coefficients by thresholding, and finally reconstruct the image by using the derived wavelet coefficients and make the difference between the original image to obtain the PRNU noise image. The process of PRNU extraction is illustrated in Fig 4.

#### Construction of the PRNU stream network

Since the traditional methods for feature extraction are often based on manually set rules and algorithms, they cannot effectively extract these complex feature patterns, we introduce a network to perform end-to-end extraction on PRNU images. By constructing a network model for the automatic learning and analysis of PRNU features, the model can effectively extract and analyze the global feature differences between real and fake faces. This enables the network to more accurately capture complex patterns and structural information in fake faces for identification.

The blue box in Fig 3 represents the structure of the PRNU stream network. Input images sequentially pass through three feature extraction modules, each comprising a convolutional layer and a max-pooling layer. Each feature extraction module introduces the non-linear activation function ReLU, improving the fitting capability of the network. The PRNU stream network constitutes a crucial component in the construction of the dual-stream network. Integrating PRNU features with CNN achieves a synergistic effect, facilitating the learning of more enriched feature representations. This integration holds significant importance in enhancing the generalization capability of the overall model.

### Quaternion RGB stream network

The PRNU stream can detect fake faces with global facial manipulation. However, the PRNU extraction relies on the statistical characteristics of the entire image, its perceptual sensitivity to subtle local changes is insufficient, this deficiency in sensitivity results in a limited capability to detect local manipulated faces. To address this issue, we designed a quaternion RGB Stream for the detection of face manipulation at the local level.

The quaternion RGB Stream integrates the operational characteristics of quaternions, and represents the face as a quaternion three-dimensional matrix, by leveraging the rich information representation capacity of quaternion structures, the network can accurately capture and analyze local manipulation in fake faces, including color, background boundaries, and spatial structures of individuals. It overcomes the defects of the conventional CNN, specifically the weakness in handling inter-channel information and color correlation, which are not conducive to extracting the differential features between real and fake images, enhances recognition accuracy in detecting local manipulation. In addition, rotation invariance and scale invariance of quaternion ensure robustness in the face of image translation and scale transformation, so that the quaternion CNN can deal with rotation and scale changes more stably when processing faces, thus improving the robustness of the model.

The red box in Fig 3 represents the structure of the quaternion RGB stream, comprising image dimension increase module, feature extraction module. In the image dimension increase module, we elevate the image to four dimensions, to fit the quaternion representation. Each part of the feature extraction module consists of a quaternion convolution [39] layer and a quaternion maximum pooling layer, enhanced with activation functions and batch normalization operations to boost feature expression capabilities.

#### Image dimension increase module

To ensure the images align with the computational requirements of the quaternion CNN, we introduce an image augmentation module. By introducing quaternion, the three channels are extended to four dimensions, the quaternion CNN uses the structural characteristics of one real part and three imaginary parts of quaternion, the interaction and correlation between different channels are described comprehensively. This operation amplifies the difference between real and fake faces and captures the complex local feature changes of fake faces more accurately. As a further extension of the concepts of real and complex numbers, the quaternion structure consists of a real part and an imaginary part composed of three elements. Specifically, a quaternion can be expressed as Eq 3,

$$\begin{array}{c} F=H+F_{r}i+F_{g}j+F_{b}k\;\;\text{(s.t.}\;H,F_{r},F_{g},F_{b}\in R) \end{array}$$ (3)

where *H*,*F*_*r*_**i**,*F*_*g*_**j**,*F*_*b*_**k** are all real numbers and belong to the real number field R. **i**, **j**, **k** are three independent imaginary units that follow specific quaternion algebraic rules, namely **i**^2^ = **j**^2^ = **k**^2^ = **i** **j** **k** = -1. In the construction of quaternions, *H* represents the real part, while *F*_*r*_**i** + *F*_*g*_**j** + *F*_*b*_**k** forms its imaginary part. If H is equal to 0, we can say that *F* is a pure quaternion. Like the real and complex number systems, the quaternion system also operates independently, as shown in Eqs 4, 5, 6 and 7 below. These laws allow us to perform mathematical operations within the framework of quaternions, thus extending the representation of mathematics in multidimensional Spaces.

Addition or subtraction:

$$\begin{array}{c} F_{1}\pm F_{2}=(H_{1}\pm H_{2})+(F_{r1}\pm F_{r2})i+(F_{g1}\pm F_{g2})j+(F_{b1}\pm F_{b2})k \end{array}$$ (4)

Conjugation:

$$\begin{array}{c} F^{*}=H-F_{r}i-F_{g}j-F_{b}k \end{array}$$ (5)

Norm:

$$\begin{array}{c} \left|F\right|=\sqrt{FF^{*}}=\sqrt{H^{2}+{F_{r}}^{2}+{F_{g}}^{2}+{F_{b}}^{2}} \end{array}$$ (6)

Quaternion multiplication:

$$\begin{array}{c} \begin{array}{cc} F_{1}F_{2}= & \left(H_{1}H_{2}-F_{r1}F_{r2}-F_{g1}F_{g2}-F_{b1}F_{b2}\right)\\ & +(H_{1}F_{r2}+F_{r1}H_{2}+F_{g1}F_{b2}-F_{b1}F_{g2})i\\ & +(H_{1}F_{g2}-F_{r1}F_{b2}+F_{g1}H_{2}+F_{b1}F_{r2})j\\ & +(H_{1}F_{b2}+F_{r1}F_{g2}-F_{g1}F_{r2}+F_{b1}H_{2})k \end{array} \end{array}$$ (7)

#### Quaternion convolution layer

Different from CNNs which use a real matrix as the measuring unit of feature mapping, each feature mapping in quaternion CNN is a quaternion matrix. Therefore, we design a quaternion convolution module to reconstruct the eigenmaps in the form of a quaternion matrix. Quaternion RGB streams take into account the correlation between different channels and extend each element in the feature map from a one-dimensional real number to a four-dimensional vector. This extension requires quaternion vectorization to adapt to the operation rules of convolutional neural networks. When performing a quaternion convolution operation, the multiplication of the real numbers is replaced by quaternion multiplication, imitating the traditional product operation. Assuming the input quaternion is represented as *X* = *x*_0_ + *x*_1_**i** + *x*_2_**j** + *x*_3_**k** and the quaternion of the convolution kernel is represented as *W* = *w*_0_ + *w*_1_**i** + *w*_2_**j** + *w*_3_**k**, the calculation of quaternion convolution can be expressed as Eq 8,

$$\begin{array}{c} \begin{array}{cc} W⊗X= & \left(w_{0}x_{0}-w_{1}x_{1}-w_{2}x_{2}-w_{3}x_{3}\right)\\ & +(w_{0}x_{1}+w_{1}x_{0}+w_{2}x_{3}-w_{3}x_{2})i\\ & +(w_{0}x_{2}-w_{1}x_{3}+w_{2}x_{0}+w_{3}x_{1})j\\ & +(w_{0}x_{3}+w_{1}x_{2}-w_{2}x_{1}+w_{3}x_{0})k \end{array} \end{array}$$ (8)

where ⊗ represents the quaternion convolution operation, each part of the input and convolution kernel is multiplied separately, and the sum is calculated according to the quaternion multiplication rules. The convolution formula can be simplified by combining similar terms, and the result is a quaternion matrix, and the real and imaginary parts can be obtained by adding or subtracting four ordinary convolution respectively. This method greatly simplifies the quaternion convolution operation and makes the neural network architecture easier to implement.

**Fig 5:**
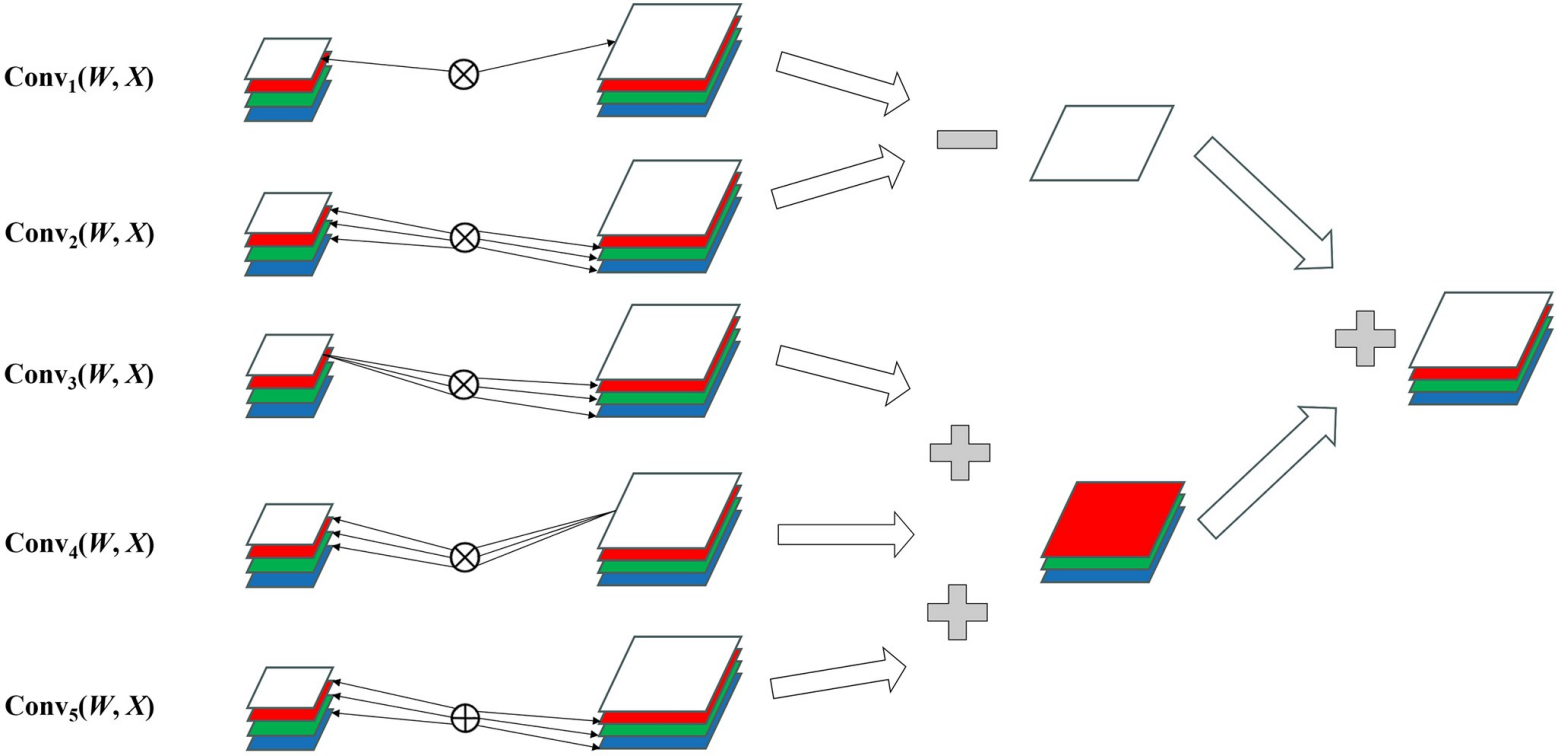
Schematic diagram of a quaternion convolution operation.

Fig 5 illustrates the process of quaternion convolution, where the operator ⊗ represents the standard convolution operation, and ⊕ denotes a special convolution form that involves pairing through cross-product operations, the cross product of Conv5 can be expressed as 9. In Conv1 and Conv2, the kernel components are element-wise multiplied and summed with the input components, ultimately yielding the real part of the quaternion. Conv3 and Conv4 demonstrate the convolution process between the kernel real part and the input imaginary part, as well as the convolution between the input real part and the kernel imaginary part, respectively. In Conv5, the kernel and input imaginary parts are paired through vector cross-product, resulting in six standard convolution outcomes, and their sum yields the final imaginary part of the quaternion. Through this concise and efficient quaternion convolution approach, the network can effectively leverage information from each channel, enhancing sensitivity and accuracy in detecting locally manipulated fake faces.

$$\begin{array}{c} \left[\begin{array}{c} w_{1}\\ w_{2}\\ w_{3} \end{array}]⊕[\begin{array}{c} x_{1}\\ x_{2}\\ x_{3} \end{array}]=[\begin{array}{c} w_{2}x_{3}-w_{3}x_{2}\\ w_{3}x_{1}-w_{1}x_{3}\\ w_{1}x_{2}-w_{2}x_{1} \end{array}\right] \end{array}$$ (9)

#### Quaternion max-pooling layer

**Fig 6:**
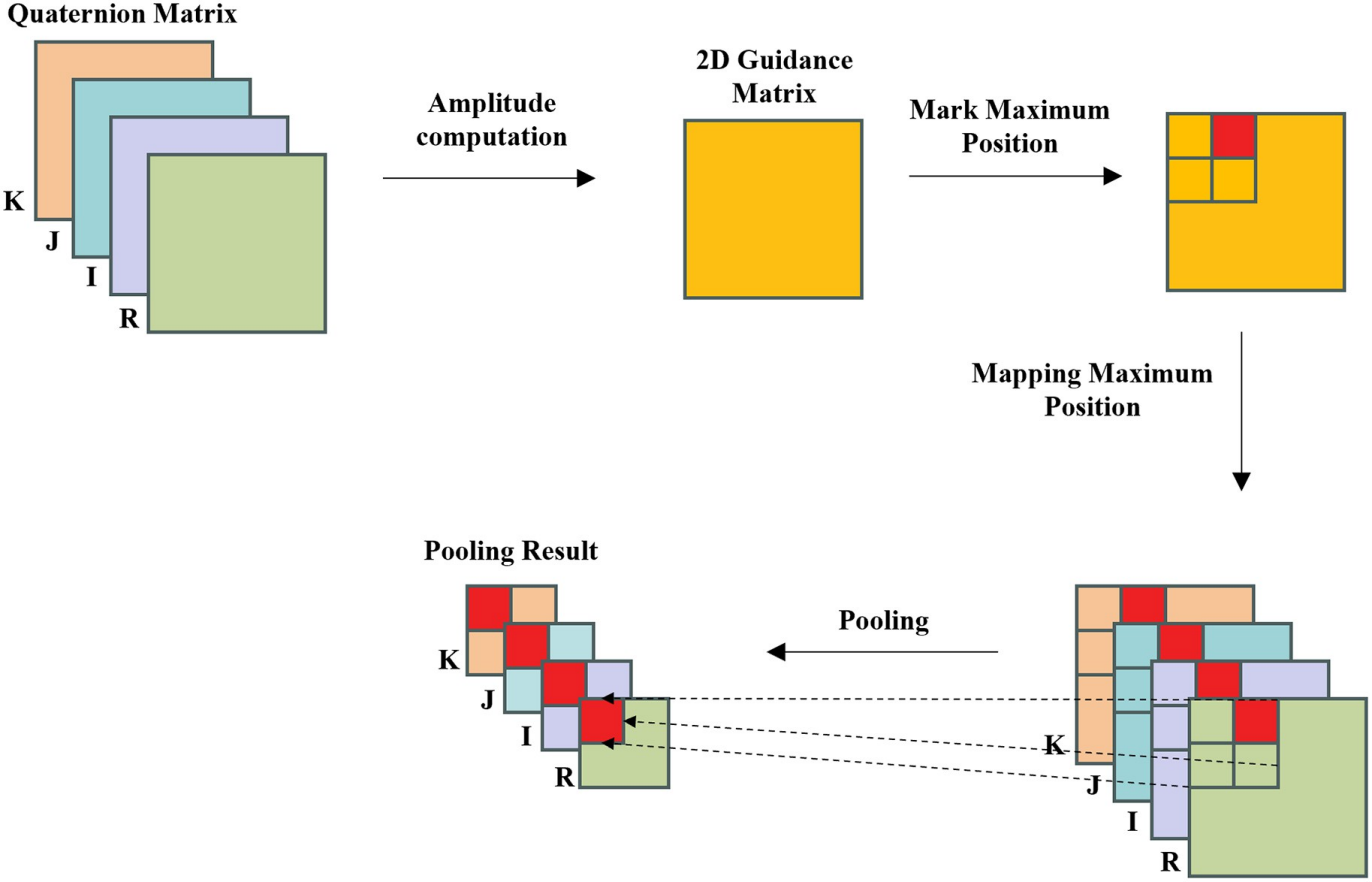
Diagram of the quaternion pooling process.

Common pooling operations include max pooling and average pooling, etc. In the context of detecting local manipulation, max pooling exhibits higher sensitivity to local features compared to average pooling. Max pooling selects the most significant feature values within a local region, emphasizing subtle changes in that region. When dealing with locally manipulated fake faces, the max pooling mechanism can more accurately capture the differences between the manipulated region and its surrounding areas, enhancing sensitivity to manipulated faces. However, directly applying traditional CNN max pooling can lead to confusion in quaternion data. Therefore, we utilize the guiding matrix of quaternion matrices to direct the pooling operation and simplify various parts of the quaternion matrix based on the results of the guiding matrix. This process relies on quaternion statistical features and performs overall pooling on the quaternion matrix based on the positions of the maximum values in each block of the two-dimensional guiding matrix. The quaternion max pooling process is illustrated in Fig 6. Specifically, we first calculate the magnitude of the quaternion matrix, which reflects the distribution of image energy, and can build a stable guide matrix for the quaternion matrix without changing the spatial information. A guide matrix is a two-dimensional matrix in which each element corresponds to the location of the maximum amplitude of a region in the quaternion matrix. Based on the guide matrix, we determine the maximum position of each quaternion block, and combine the corresponding values of the real and imaginary parts to complete the maximum pooling of the entire quaternion matrix. This process not only preserves the spatial and energy distribution characteristics of the quaternion matrix, but also ensures the consistency between the various parts, avoiding the data inconsistency problem caused by directly pooling each part of the quaternion separately.

## Experiments

This section comprises the fundamental settings, training methodology, and the employed face datasets in our experiments. Through a series of experiments, we validate the significant efficacy of the detection network based on the dual streams of PRNU and quaternion RGB in the task of discriminating real and fake faces, achieving a remarkable detection accuracy of 96.81%.

### Experimental setups

**Fig 7:**
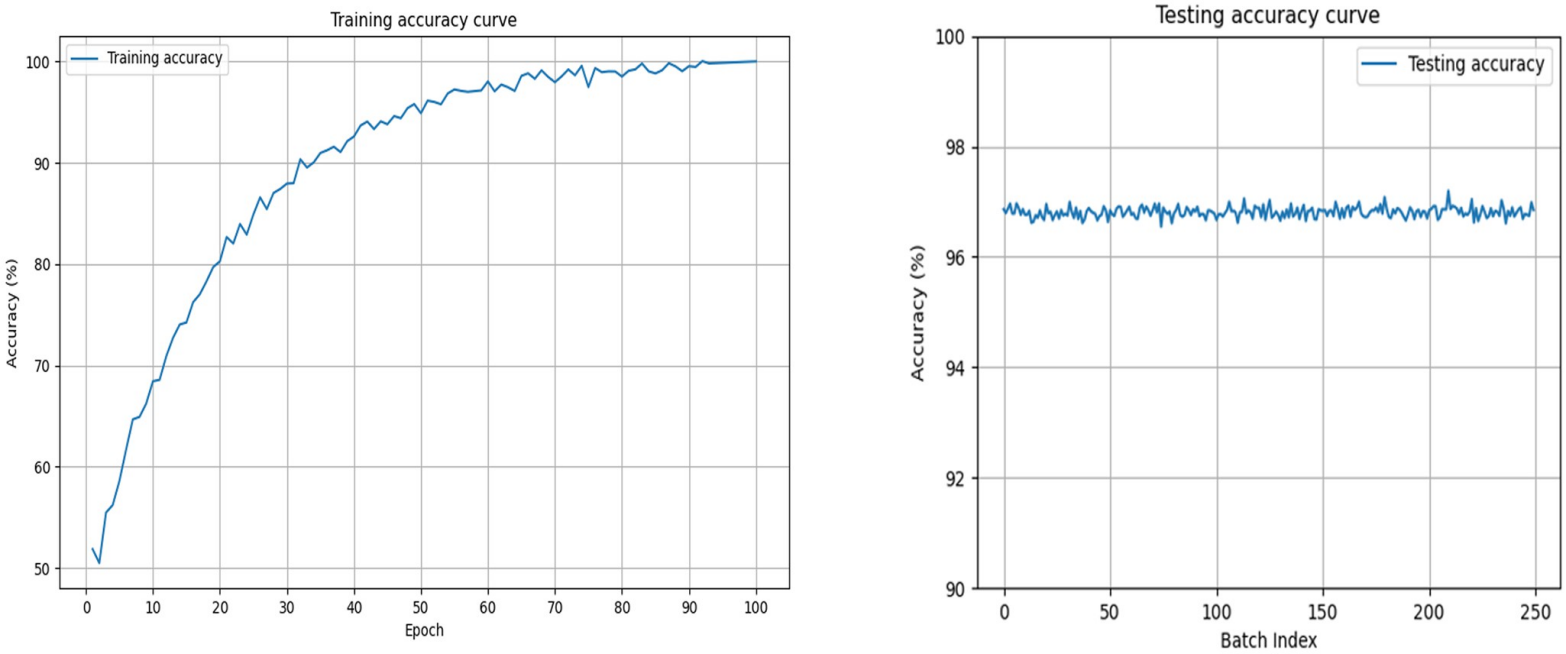
Accuracy graphs for training and testing.

During the training process, we used the Adam optimizer [40] and set the hyperparameters, including a batch size of 16 and a total of 100 training periods. In order to better control the learning rate and improve the generalization ability of the model, we adopt the learning rate attenuation strategy: in the first 50 training periods, the learning rate is set to 0.001; In the last 50 training periods, the learning rate was reduced to 0.0001. We use the classical cross-entropy function to measure the difference between the prediction of the model and the real label. Fig 7 shows the variation in accuracy over the course of training, as well as the stability of accuracy over the test set. As can be seen from Fig 7, with the progress of training, the training accuracy rate presents a trend of fluctuation and increase. Around the 93rd training period, the training accuracy reached a steady state and reached 100% accuracy. This shows that our model has sufficiently learned the features in the training data and is able to accurately classify the input true and false faces. The accuracy graph on the test set shows the stability of the model on different batches of data. It can be seen that the accuracy of the test fluctuates only slightly between batches, with an average accuracy of 96.81%. This proves that our method can reliably detect fake faces.

We use the FFHQ dataset [2] and the Celeb-DF-V1 dataset [41] as experimental datasets. The FFHQ dataset comprises 70,000 facial images, covering diverse age groups, genders, ethnicities, and poses. Celeb-DF-V1 is a publicly available dataset widely used for deep learning models, specifically designed for tasks like facial attribute forgery detection and Deepfake recognition. The fake face dataset comes from images generated by StyleGAN cite ref-1 and Celeb-DF-V1. In the experiments, we standardize the image dimensions to 256x256, this approach facilitates the model in processing and comparing features between real and fake faces in a uniform manner. In the ablation experiment, network layer comparison experiment, and comparison experiment, the training uses real face data from FFHQ and Celeb DF-V1, with 5000 real faces each. The fake face data used during training comes from the fake faces in StyleGAN and Celeb DF-V1, and the number is the same as the real face data. In the experiments, the model is evaluated on a test set containing 2000 real faces and 2000 fake faces, with test data sourced from different images in the same dataset as the training data. In all experiments, we use accuracy to evaluate network performance, as defined by the formula 10,

$$\begin{array}{c} Accuracy=\frac{T_{P}+T_{N}}{P+N} \end{array}$$ (10)

where *P* and *N* represent the quantities of fake and real faces in the experiment, *T*_*P*_ and *T*_*N*_ represent the quantities of correctly classified fake and real faces, respectively.

### Experimental results

#### Ablation study

The experimental results are shown in Table 1. It can be seen that the performance of PRNU feature extraction using Haar wavelet is significantly improved compared with conventional noise reduction extraction methods, which indicates that Haar wavelet can better capture effective feature differences between real faces and fake faces, and improve the accuracy and reliability of false human detection. However, the detection accuracy of PRNU stream and quaternion RGB stream are both below 95%, which indicates that a single network stream cannot effectively distinguish the global and local manipulated faces at the same time. In the third experiment, we fuse the quaternion RGB stream and the PRNU stream to construct a dual-stream detection network, achieving a final accuracy of 96.81%. From the aforementioned experimental results, it can be observed that each module and branch in the proposed dual-stream detection network is necessary and reasonable. The comprehensive use of PRNU features and quaternion RGB features can effectively detect global and local manipulated faces, and cope with various forms of face tampering.

**Table 1:** Ablation experiment results.

Model	Accuracy(%)
PRNU Stream(Gaussian noise reduction)	83.24
PRNU Stream([30])	86.81
PRNU Stream(Haar wavelet)	88.75
Quaternion RGB Stream	90.13
Quaternion RGB Stream + PRNU Stream(Haar wavelet)	**96.81**

#### The performance of hypercomplex numbers in fake face forensics

To further explore the application of hypercomplex numbers in the field of fake face forensics, we tested the recognition accuracy of quaternion, octonion, and sedenion as well as the traditional CNN network. Considering the rigor of the experiment, only locally tampered fake faces were selected for fake faces. The convolutional kernel pooling design of octonion and sedenion CNN was based on our quaternion CNN as the template.

The experimental results are shown in Table 2. As can be seen from the table, compared with the traditional CNN structure, the performance of quaternion CNN has been significantly improved, which indicates that quaternion CNN can process the RGB color channels closely, effectively capture the interdependence between the color channels, and construct comprehensive information about the local tampering area of the face, to effectively detect the subtle color inconsistencies in the local tampering face. Because the traditional CNN ignores the correlation between color channels, it is not sensitive enough to the change of local tampering regions, and the detection accuracy is low. Compared with quaternion CNN, the detection accuracy of octonion CNN also increases while that of sedenion CNN decreases. We consider that octonion and sedenion provide higher dimensions, and appropriately increasing the dimension of hypercomplex numbers can improve the detection accuracy of fake faces. However, excessive dimensions may introduce redundant features in fake faces. This redundant information not only does not improve the detection effect, but also may make the model more sensitive to irrelevant or noisy features in the face, and cannot effectively match with the local tamper region. In addition, although the accuracy of octonion CNN is improved compared with quaternion CNN, the calculation complexity is increased due to the high dimension of octonion and sedenion. This will affect the real-time and efficiency of the model, so we still choose the quaternion CNN as the branch of the two-stream network.

**Table 2:** Comparative experiment of accuracy of different nions.

Nions	Accuracy(%)
3	94.15
4	96.47
8	**96.83**
16	95.56

#### Comparison of network layer groups

The primary objective of the proposed dual-stream CNN is to effectively distinguish between real faces and fake faces. To achieve this, we conduct the network layer number experiment. By varying the number of network layers, we observe and analyze the performance changes of the model at different depths. This helps us identify the optimal network structure to enhance the robustness of the model while avoiding overfitting and excessive complexity. We compare networks with different depths and find that a 4-layer network structure yields the best performance for constructing the dual-stream CNN. The experimental results are presented in Table 3. From the results, it can be seen that when the network depth is 3 layers, the detection accuracy is only 93.72%. It suggests that if the depth is shallow, the network can not capture features adequately, limiting the model’s performance. When the network depth is 5 layers, the performance is almost the same as the 4-layer network. However, a 5-layer network has more layers and parameters, requiring more computational resources and time for training. Considering this trade-off, we decide to use a 4-layer network to balance computational resource consumption and task requirements, thereby completing the construction of the final model.

**Table 3:** Comparison of network layer group numbers.

Model	Accuracy(%)
3	93.72
4	96.81
5	**96.87**

#### Comparison experiments

In comparison experiments, we compare the performance of the proposed dual-stream network with other solutions. For the comparative experiment, we select several popular network architectures and methods, including those based on traditional image forensics, deep learning, and the latest research findings in fake face detection. The results are presented in Table 4, and through the comparative analysis, it is evident that our proposed dual-stream network outperforms other approaches in the task of detecting fake faces. This achievement is attributed to the ability of the dual-stream network to effectively leverage the fusion of PRNU and quaternion RGB features, comprehensively considering the feature differences between globally and locally manipulated regions. This ensures that the network can efficiently learn and capture the feature patterns of fake faces, thereby enhancing detection accuracy and reliability.

**Table 4:** Comparison experiments.

Model	Accuracy(%)
Nataraj’s method [9]	93.49
Wang’s method [10]	94.08
Wang’s method [11]	92.01
Our method (Two-stream)	**96.81**

#### Generalization experiments

The generalization experiment selects the same training data as the above experiments and evaluates the generalization performance of the model on six fake face datasets: StyleGAN II [42], Celeb DF-V2 [43], StyleGAN III [44], FaceForensics++ [45], BigGAN [46], and DFDC [47]. The model is evaluated on a test set containing 2000 real faces and 2000 fake faces. The real face data used in the testing comes from the CelebA dataset and the real faces captured from the aforementioned video dataset to ensure the rigor of the generalization experiment.

In this experiment, We conducted network generalization experiments and comparisons on three sets of fake face datasets, and all images had the same resolution. The experimental results are presented in Table 5. The experimental results show that our dual stream network demonstrated satisfactory performance on three sets of fake face datasets. This is mainly attributed to the effective generalization ability of our model to fake face data from different sources. The PRNU stream captures the characteristic patterns of global tampering, while the quaternion RGB stream focuses more on capturing and analyzing the fine features of local regions in the images, the organic fusion of global and local features allows our model to comprehensively understand the feature differences in fake faces, thereby improving detection accuracy and robustness. Additionally, we carefully consider the diversity and scale of the dataset during network training, enhancing the generalization performance of the model to adapt better to fake faces from different sources, thereby increasing the practicality and reliability of the network in real-world applications.

**Table 5:** Generalization experiments.

Datasets	Methods	Accuracy(%)
StyleGAN II and Celeb-DF-V2	Nataraj’s method [9]	78.26
	Wang’s method [10]	77.81
	Wang’s method [11]	75.32
	Our method (Two-stream)	**82.25**
StyleGAN III and FaceForensics++	Nataraj’s method [9]	75.95
	Wang’s method [10]	76.63
	Wang’s method [11]	72.42
	Our method (Two-stream)	**81.58**
BigGAN and DFDC	Nataraj’s method [9]	79.84
	Wang’s method [10]	80.67
	Wang’s method [11]	77.30
	Our method (Two-stream)	**85.06**

## The difference between quaternion convolution and traditional convolution

**Fig 8:**
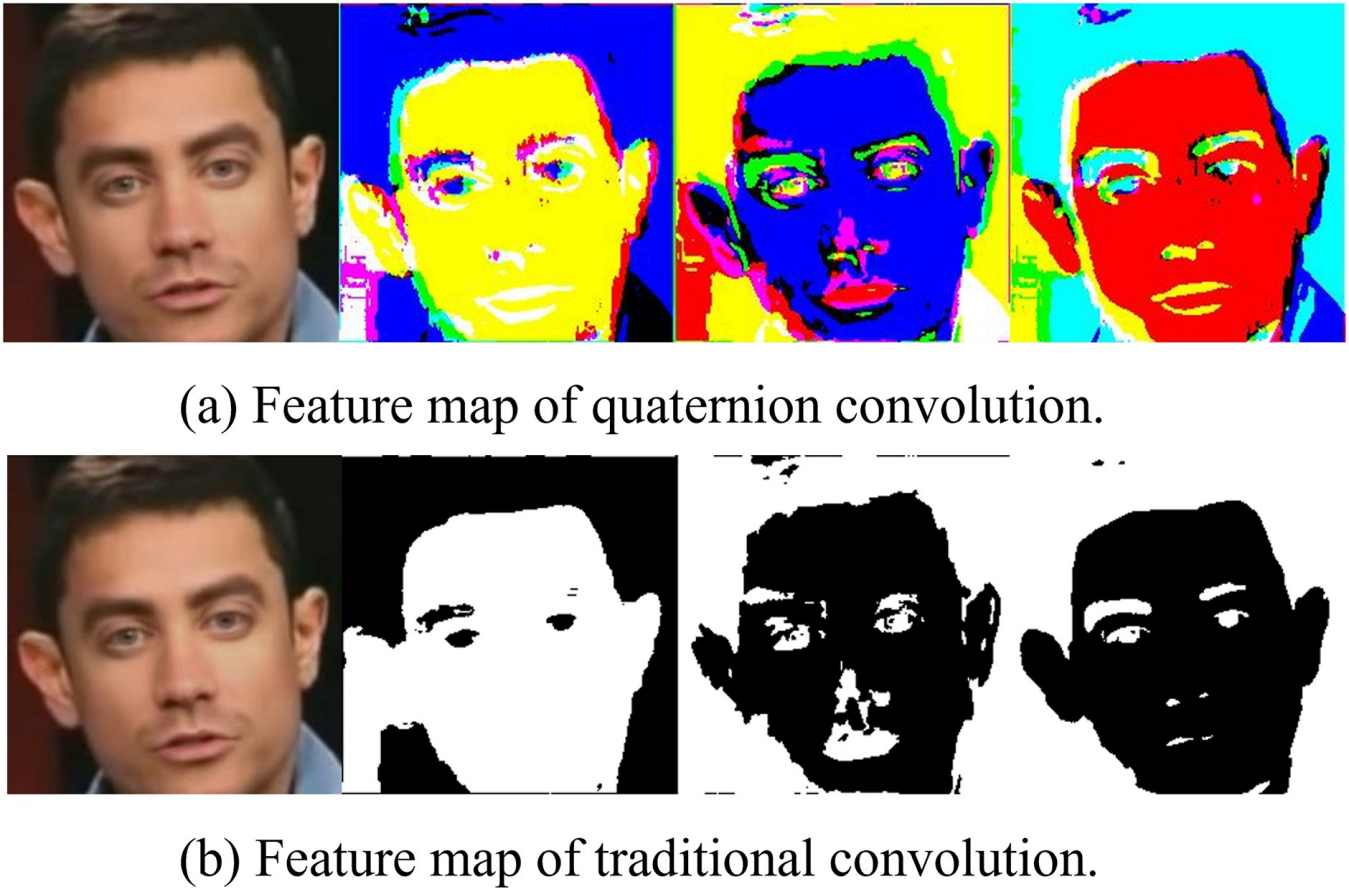
Feature map of quaternion and traditional convolution.

To facilitate the understanding of the advantages of quaternion convolution and reflect the difference between it and traditional convolution, we visualized the feature maps of the two respectively. As shown in Fig 8, it can be seen that quaternion convolution is more sensitive in processing fake faces by its sensitivity to color information and deep fusion ability of multi-channel features. Quaternion CNN can not only capture the features of the local tampered region in the fake face, but also provide additional dimensions for the representation of image features, enabling quaternion CNN to process the color of the face image and the potential image texture and other information, so it can clearly distinguish the subtle color changes in the face image and the differences in the local tampered region. More discriminant information for fake face detection. In contrast, the feature representation of traditional CNN is limited to feature extraction at the spatial level, which fails to make full use of the dependency between color channels and leads to the lack of contrast of color differences in color information, resulting in insufficient feature expression ability.

## Visualization of features in quaternion RGB

**Fig 9:**
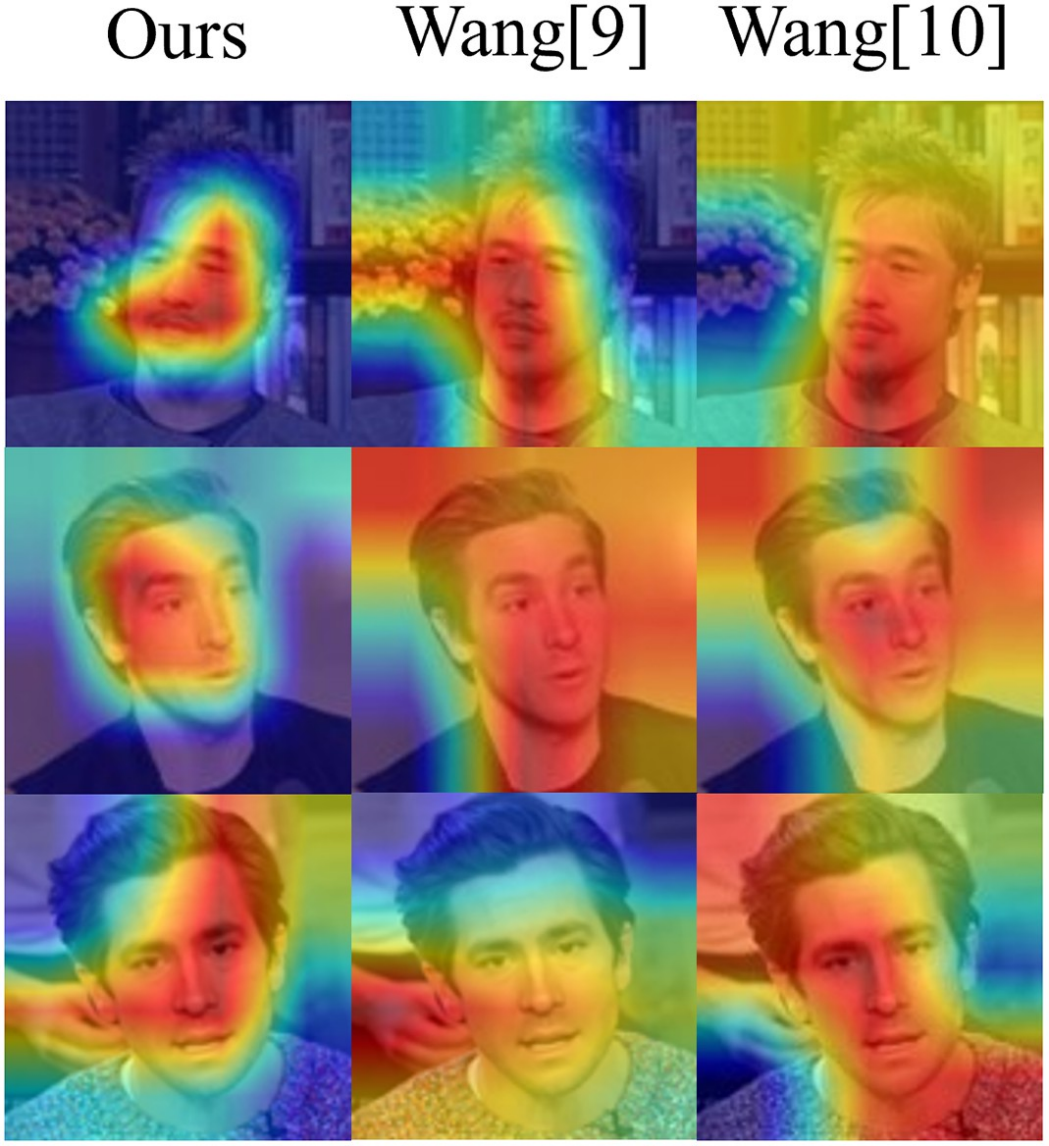
Heat map display of locally tampered fake faces.

Fig 9 illustrated the regions that the models focus on when detecting fake faces. The quaternion RGB stream demonstrates heightened sensitivity to local features, allowing it to hone in on more precise areas within the image. This capability enables the stream to more accurately capture the characteristics of locally tampered fake faces, distinguishing these manipulated regions from the authentic ones. By emphasizing and enhancing the network’s attention on these modified local features, the model can effectively and precisely identify tampered sections within the facial image. Consequently, this improved focus on subtle, localized discrepancies allows for more accurate and reliable detection of fake faces with local tampering.

## Visualization of attention area of single stream branch and dual stream network

**Fig 10:**
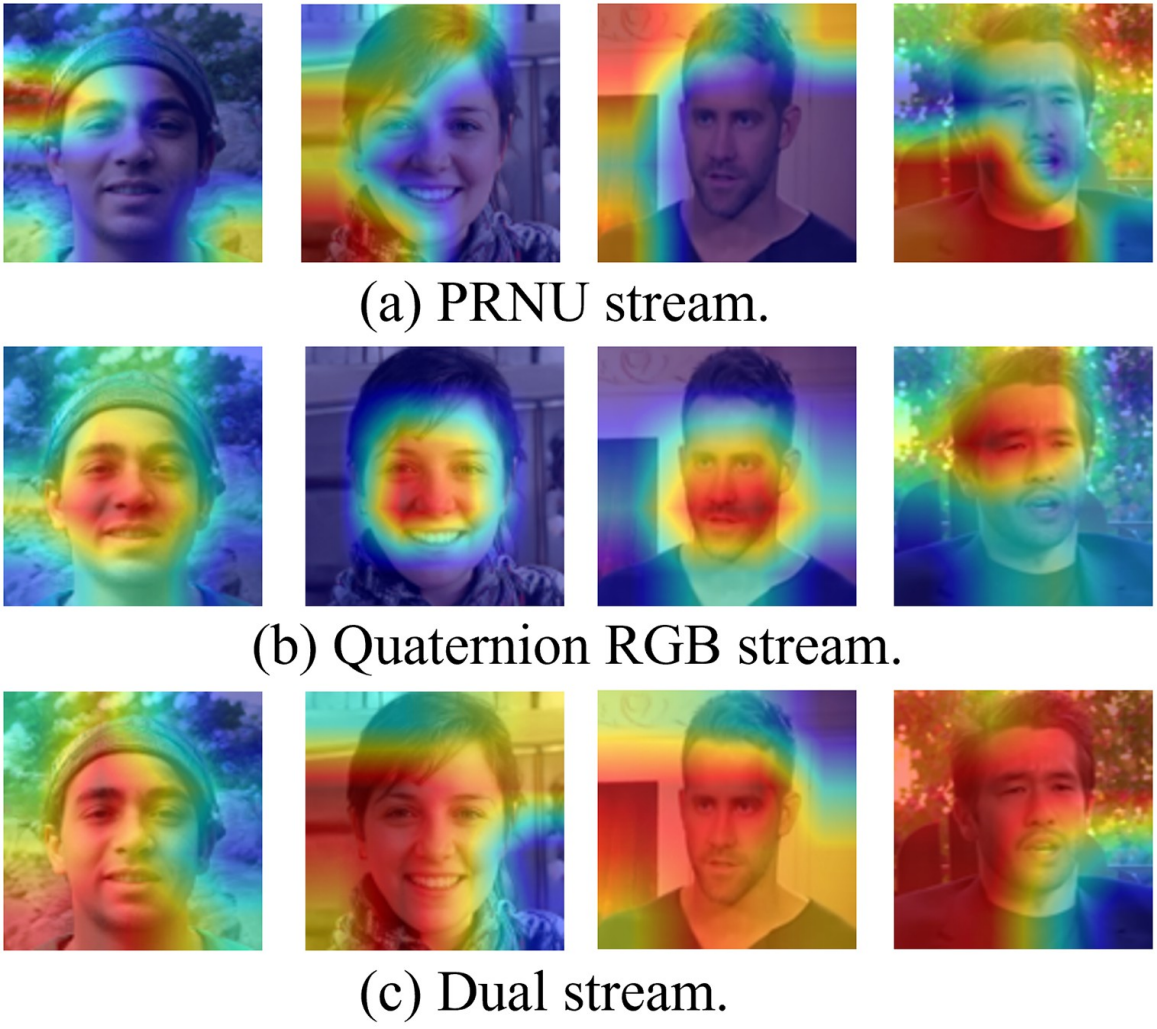
The visualization of attention regions of PRNU stream, quaternion RGB stream and the dual stream we proposed.

Specifically, we demonstrated in the experiment the heat maps generated by PRNU stream network and quaternion RGB network when processing fake faces, to observe their respective capture of facial tampering features. Subsequently, we compared the results of these two single-stream networks with the heatmap of the dual stream network, as shown in Fig 10. It can be seen that the quaternion RGB stream has higher sensitivity to local features and can focus on finer areas of the face. PRNU stream is more sensitive to global features and can capture the overall consistency and texture features of the faces, such as background, edges, etc., thus better characterizing the characteristic changes of the face under global tampering. The dual stream network we propose exhibits simultaneous sensitivity to global and local tampering features, capturing both the overall consistency and texture changes of the image, as well as focusing on subtle local areas to accurately detect local tampering features, achieving complementary detection under global and local tampering, and thus exhibiting stronger accuracy in detecting fake faces.

## Speed in real-time systems

All of the experiments are implemented using PyTorch, where we run our network and other experiments on a 24GB GeForce RTX 4090, 3.79GHz AMD Ryzen 5 CPU, and 32GB RAM. Table 6 shows the calculation time of the respective methods, although the proposed method is not the fastest among the evaluation methods, it is still fast, and considering its balance of accuracy and real-time, we believe that it still has good performance in practical applications.

**Table 6:** Computational time for training and testing.

Method	Train computational time(s)	Test Computational time(s)
Nataraj’s method [9]	**0.0041**	**0.0022**
Wang’s method [10]	0.0049	0.0026
Wang’s method [11]	0.0046	0.0023
Our method (Two-stream)	0.0053	0.0027

In addition, In Table 7, we test the detection speed of quaternion, octonion, and sedenion CNN in the real-time system, which is used to comprehensively sum up the performance of our method. Compared with quaternion, octonion and 16-nion introduce higher dimensions, which can theoretically capture more complex image features, but at the same time increase the computational complexity and resource requirements of the model. This will affect the real-time and efficiency of the model. In practical application scenarios such as social media content review and false information dissemination monitoring, fake face detection must have efficient real-time response capability. The efficiency of quaternion CNN in combining multi-channel data and the high sensitivity to subtle tampering can effectively reduce the detection time, although not the fastest of all methods, but still can ensure that the system can respond quickly to meet the large-scale and diverse challenges of fake face generation.

**Table 7:** Calculation times for training and testing with different nions.

Nions	Train computational time(s)	Test Computational time(s)
3	**0.0024**	**0.0011**
4	0.0027	0.0013
8	0.0032	0.0017
16	0.0040	0.0021

## Disscussion

We propose a fake face detection network based on PRNU and quaternion RGB to address the limitations of conventional methods in processing facial images generated by a single AIGC technology. By fusing the differences in global and local features between real and fake faces through a dual-stream network, our method can effectively detect AIGC faces that have been tampered with both globally and locally, thus standing out among existing technologies. Currently, our research mainly focuses on static images and has not yet considered the detection of fake facial videos. In videos, in addition to considering the authenticity of each frame of the image, it is also necessary to consider dynamic information such as inter-frame continuity and motion trajectories, which puts higher demands on the accuracy of facial feature extraction and the learning performance of the network itself. In the future, we will study how to detect dynamic fake faces under various tampering methods.

## Conclusion

In this paper, the fake face detection network based on PRNU and quaternion RGB we proposed demonstrates significant advantages in both global and local tampering detection. By leveraging the distinctive texture features of PRNU and the multi-dimensional color information from quaternion RGB, our model can accurately capture subtle differences in fake faces. Experimental results indicate that our approach exhibits outstanding generalization capabilities across multiple datasets. Our method not only effectively distinguishes between real and fake faces but also demonstrates stable performance across different types of fake faces. These findings provide valuable insights for future research and improvements, offering robust support in addressing the evolving landscape of fake face technologies and adversarial attacks.
